# LINC00668在肺鳞癌中高表达并促进肿瘤细胞的侵袭转移

**DOI:** 10.3779/j.issn.1009-3419.2022.102.05

**Published:** 2022-04-20

**Authors:** 博 袁, 洋 陈, 竞妍 袁, 丽忠 曾, 拴盈 杨

**Affiliations:** 710004 西安，西安交通大学第二附属医院呼吸与危重症医学科 Department of Pulmonary and Critical Care Medicine, The Second Affiliated Hospital of Xi'an Jiaotong University, Xi'an 710004, China

**Keywords:** 肺肿瘤, LINC00668, 转移, 侵袭, Lung neoplasms, LINC00668, Migration, Invasion

## Abstract

**背景与目的:**

由于缺乏有效的治疗措施，肺鳞癌(lung squamous cell carcinoma, LUSC)仍是制约非小细胞肺癌(non-small cell lung cancer, NSCLC)5年生存率的重要影响因素。长链非编码RNA 00668(LINC00668)参与调控多种肿瘤的发生发展，但其在LUSC中的研究尚不完善。本研究旨在探讨LINC00668在NSCLC特别是LUSC中的预后价值及生物学功能。

**方法:**

本研究利用癌症基因组图谱(The Cancer Genome Altas, TCGA)数据库分析了LINC00668在NSCLC特别是LUSC中的表达模式及其与患者临床特征和预后的关系; 并通过体外实验探索了其在LUSC细胞中的功能。

**结果:**

LINC00668在LUSC组织中高表达，并与患者的肿瘤原发灶-淋巴结-转移(tumor-node-metastasis, TNM)分期显著相关。同时，LINC00668在吸烟患者中的表达量明显升高，并与吸烟LUSC患者的总生存期(overall survival, OS)显著相关。体外实验显示，相较于正常支气管上皮细胞和癌旁组织，LINC00668在LUSC细胞系和组织样本中表达量显著升高; 同时，在LUSC细胞系中敲低LINC00668可有效抑制细胞的侵袭转移能力。

**结论:**

LINC00668可能与肺鳞癌发生发展密切相关，抑制LINC00668可在一定程度上减少LUSC的转移。

## Abstract (en)

**Background and objective:**

A lack of effective treatment for lung squamous cell carcinoma (LUSC) makes it an important factor restricting the 5-year survival rate of non-small cell lung cancer (NSCLC). Long non-coding RNA 00668 (LINC00668) was reported to play crucial regulatory roles in the tumorigenesis and progression of various cancers; however, its role in LUSC is unclear. The aim of this study was to investigate the prognosis value and biological function of LINC00668 in NSCLC, especially in LUSC.

**Methods:**

The expression pattern of LINC00668 and its relationship with clinical characteristics and prognosis of patients were investigated in the NSCLC especially LUSC based on The Cancer Genome Altas (TCGA) database. Its function in LUSC cells was explored *in vitro*.

**Results:**

LINC00668 expression was significantly up-regulated in LUSC patients and high expression level of LINC00668 was associated with advanced tumor-node-metastasis (TMN) stage. Moreover, the expression of LINC00668 significantly increased in smoking patients, and was a prognostic indicator for overall survival (OS) of smoking patients with LUSC. *In vitro* experiments showed that LINC00668 has significantly higher expression level in LUSC cell lines and tissues compared to normal bronchial epithelial cell and para-tumor tissues; meanwhile, functional assay indicated knockdown of LINC00668 effectively inhibited the migration and invasion of LUSC cells.

**Conclusion:**

LINC00668 might closely relate to the development of LUSC, and inhibition of LINC00668 may reduce the metastasis of LUSC.

在全人类中，肺癌仍然是造成癌症相关死亡的主要原因之一，根据美国癌症协会的数据^[1]^统计，2020年估计有180万人(18%)死于肺癌。在所有的肺癌病例中，非小细胞肺癌(non-small cell lung cancer, NSCLC)的比例超过80%。目前最常见的NSCLC亚型包括肺腺癌(lung adenocarcinoma, LUAD)和肺鳞状细胞癌(lung squamous cell carcinoma, LUSC)，分别约占所有NSCLC病例的40%和25%-30%^[2]^。LUSC患者的总生存期(overall survival, OS)常较其他的NSCLC亚型患者短，一方面是由于确诊时患者年龄大、分期晚且常伴有转移^[3, 4]^; 另一方面，与LUAD治疗时可选择的众多小分子靶向药物相比，目前针对LUSC的治疗方法很有限，仍以化疗为主^[5]^。LUSC每年造成的死亡人数超过40万，使其成为制约NSCLC 5年生存率的重要影响因素^[6]^。因此，深入探明调控LUSC增殖或转移的相关机制并进行针对性干预，对改善患者预后十分重要。

长链非编码RNA(long non-coding RNA, LncRNAs)是最常见的不具有编码蛋白质功能的一类RNA，其序列长度超过200个核苷酸^[7]^。随着对LncRNAs生物学功能研究的不断深入，发现其可以通过表观修饰、转录及转录后调节等多种途径参与基因的调控^[8]^。LncRNAs的异常表达可引起多种人类疾病，特别是肿瘤性疾病^[9]^。据报道，LncRNAs可作为促癌或者抑癌基因参与肿瘤的发生发展^[10]^。异常表达的LncRNAs可参与包括细胞增殖、侵袭和转移、血管生成等多种生物学过程^[11, 12]^。鉴于此，LncRNAs很有可能成为肿瘤诊断的分子指标或治疗靶点，为人类肿瘤的研究提供新的方向。

LncRNA 00668(LINC00668)是一条长度为1, 751 bp的非编码RNA，定位于染色体18p11.31。LINC00668的研究在一些肿瘤中已取得初步进展，包括通过影响细胞周期或抑制凋亡促进乳腺癌细胞、肝癌细胞或结肠癌细胞的增殖，同时促进其侵袭转移^[13-16]^。An等^[17]^报道称LINC00668在NSCLC中的表达量升高，且影响LUAD细胞的增殖和转移侵袭; 然而，LUAD和LUSC除在病理学形态分化方面不同外，两种亚型在基因的表达、突变频谱、驱动基因等各方面也存在明显的差异，因此，有必要在LUAD和LUSC两种NSCLC亚型中分别研究LINC00668的预后价值及生物学功能^[18]^。

在本项研究中，我们利用癌症基因组图谱(The Cancer Genome Altas, TCGA)数据库分别分析了LINC00668在LUAD和LUSC中的表达情况及其与患者临床特征和预后的关系，并通过在体外肺鳞癌细胞系中敲低LINC00668探索其在细胞中的生物学功能。

## 1 材料与方法

### 1.1 生物信息学数据库

535例LUAD及59例正常组织和502例LUSC及49例正常组织的转录组RNA测序信息及相应的500例LUAD患者和490例LUSC患者的临床资料下载自TCGA数据库(https://gdc-portal.nci.nih.gov/)。临床资料包括性别、年龄、肿瘤原发灶-淋巴结-转移(tumor-node-metastasis, TNM)分期、吸烟情况、生存期和生存状态并总结于表 1中。*Kaplan-Meier* Plotter(Kmplot)网站(https://kmplot.com/analysis/)可以评估包括肺癌在内的21种癌症类型中54, 000种基因(mRNA, miRNA, LincRNA)对生存率的影响。

**表 1:** 肿瘤基因组图谱数据库中肺腺癌和肺鳞癌患者的临床信息 Clinical information of LUAD and LUSC patients in TCGA database

Clinical factors	LUSC	LUAD
Vital status		
Alive	282	318
Dead	212	182
Age (yr)		
≤65	189	237
> 65	300	253
Unknown	5	10
Gender		
Female	128	270
Male	366	230
Stage		
Ⅰ	242	268
Ⅱ	158	119
Ⅲ/Ⅳ	90	105
Unknown	4	8
T stage		
T1	114	167
T2	287	267
T3-T4	93	63
Unknown	0	3
N stage		
0	316	324
1	127	94
2	40	69
3	5	2
Unknown	6	11
Smoking status		
Non or ever	344	363
Current	133	119
Unknown	17	18
TCGA: The Cancer Genome Altas; LUAD: lung adenocarcinoma; LUSC: lung squamous cell carcinoma.		

### 1.2 组织收集

我们从2018年3月-2019年3月连续收集了就诊于西安交通大学第二附属医院的10例LUSC患者的肿瘤及其癌旁组织。这10例患者中有8例男性。我们的研究方案已获得西安交通大学第二附属医院伦理委员会批准同意。10例患者均提供了书面知情同意书。

### 1.3 细胞培养和转染

肺腺癌细胞系A549、H1299和肺鳞癌细胞系H2170、H226、H520以及人正常支气管上皮细胞系HBE细胞购于中国科学院细胞研究所(上海，中国)。所有细胞培养于含有10%胎牛血清和1%的青霉素及链霉素的RPMI-1640(Gibco，美国)的培养基中。所有细胞在37 ℃恒温、5%CO_2_的湿润培养箱中孵育。待细胞生长至对数期铺于6孔板进行转染。LINC00668的“短发夹”RNA(short hairpin RNA, shRNA)由上海吉凯公司设计构建，用Lipofectamine 2000(Invitrogen，美国)进行转染。转染48 h后，用嘌呤霉素筛选2代-3代，随后在显微镜下观察细胞荧光携带情况初步判断转染效率。shRNA的序列见表 2。

**表 2:** 短发夹”RNA序列 Short hairpin RNA (shRNA) sequence

ID	Sequence
LINC00668-RNAi-1-a	5’-GATCCCCCACTCCCATCCACTGTAGTGTAAACTCG AGTTTACACTACAGTGGATGGGAGTGGTTTTTGGAT-3’
LINC00668-RNAi-1-b	5’-AGCTATCCAAAAACCACTCCCATCCACTGTAGTGT AAACTCGAGTTTACACTACAGTGGATGGGAGTGGG-3’
LINC00668-RNAi-2-a	5’-GATCCCGAATCTTGGGCGGTCTGAAATCTGACTCG AGTCAGATTTCAGACCGCCCAAGATTCTTTTTGGAT-3’
LINC00668-RNAi-2-b	5’-AGCTATCCAAAAAGAATCTTGGGCGGTCTGAAATC TGACTCGAGTCAGATTTCAGACCGCCCAAGATTCGG-3’

### 1.4 实时定量逆转录聚合酶链式反应(quantitative real-time polymerase chain reaction, qRT-PCR)

组织RNA和细胞RNA分别用Fast1000和Fast200提取。总RNA用PrimerScrip^TM^ RT试剂盒(TAKARA, BIO INC)反转录为cDNA，然后按照制造商说明进行qRT-PCR反应。LINC00668及GAPDH的引物序列如表 3所示。基因的相对表达量用2^-ΔΔCq^表示^[19]^。

**表 3:** 实时定量逆转录聚合酶链式反应引物序列 Primer sequence of qRT-PCR

ID	Sequence
LINC00668	Fwd: 5’-GGGTCCAAGGGATCTGCAAG-3’
	Rev: 5’-CCGCCCAAGATTCCTCTAGC-3’
GADPH	Fwd: 5’-ATGGGGAAGGTGAAGGTCGG-3’
	Rev: 5’-GACGGTGCCATGGAATTTGC-3’
qRT-PCR: quantitative real-time polymerase chain reaction.	

### 1.5 CCK-8细胞增殖实验

收集各组细胞并接种到96孔板中，每孔约100 μL、3×10^3^个细胞。分别在铺板后24 h、48 h、72 h于每孔加入培养基总体积10%的CCK-8溶液进行实验，即每孔加入10 μL的CCK-8溶液。加入工作液后将96孔培养板重新放置于37 ℃、5%CO_2_的培养箱内继续培养约40 min，使用酶标仪在450 nm波长处检测96孔培养板各孔的吸光值即OD值，并记录实验结果。

### 1.6 划痕实验

转染后的各组细胞铺于6孔板中，待细胞培养至100%融合后，用10 μL移液管尖划痕。然后用低血清培养基(1%胎牛血清)代替完全培养基。在0 h、24 h和48 h分别对刮拭区域拍摄图像。使用Image J软件(version 1.52)计算创面面积。

### 1.7 Transwell迁移侵袭实验

收集转染后细胞，计数。将4×10^4^个H2170细胞以200 μL不含胎牛血清的培养基接种于上室，然后在下室中加入600 μL含10%胎牛血清的培养基。培养24 h后，用多聚甲醛固定细胞，用结晶紫染色。将小室上面细胞清除，显微镜下计数小室下面的细胞数。

### 1.8 统计学分析

所有统计学分析在SPSS 19.0中进行，实验数据用均数±标准差(Mean±SD)表示。计数资料采用独立样本*t*检验，计量资料采用χ^2^检验。采用*Kaplan-Meier*法估算OS，并用*Log-rank*检验进行比较。*P* < 0.05为差异具有统计学意义。

## 2 结果

### 2.1 LINC00668在肺腺癌和肺鳞癌中表达量升高

我们首先分析了TCGA数据库中535例LUAD及59例正常组织和502例LUSC及49例正常组织中LINC00668的表达量，结果显示其在LUAD及LUSC组织中的表达量均显著升高(*P* < 0.05)(图 1A，图 1B)。

**图 1:**
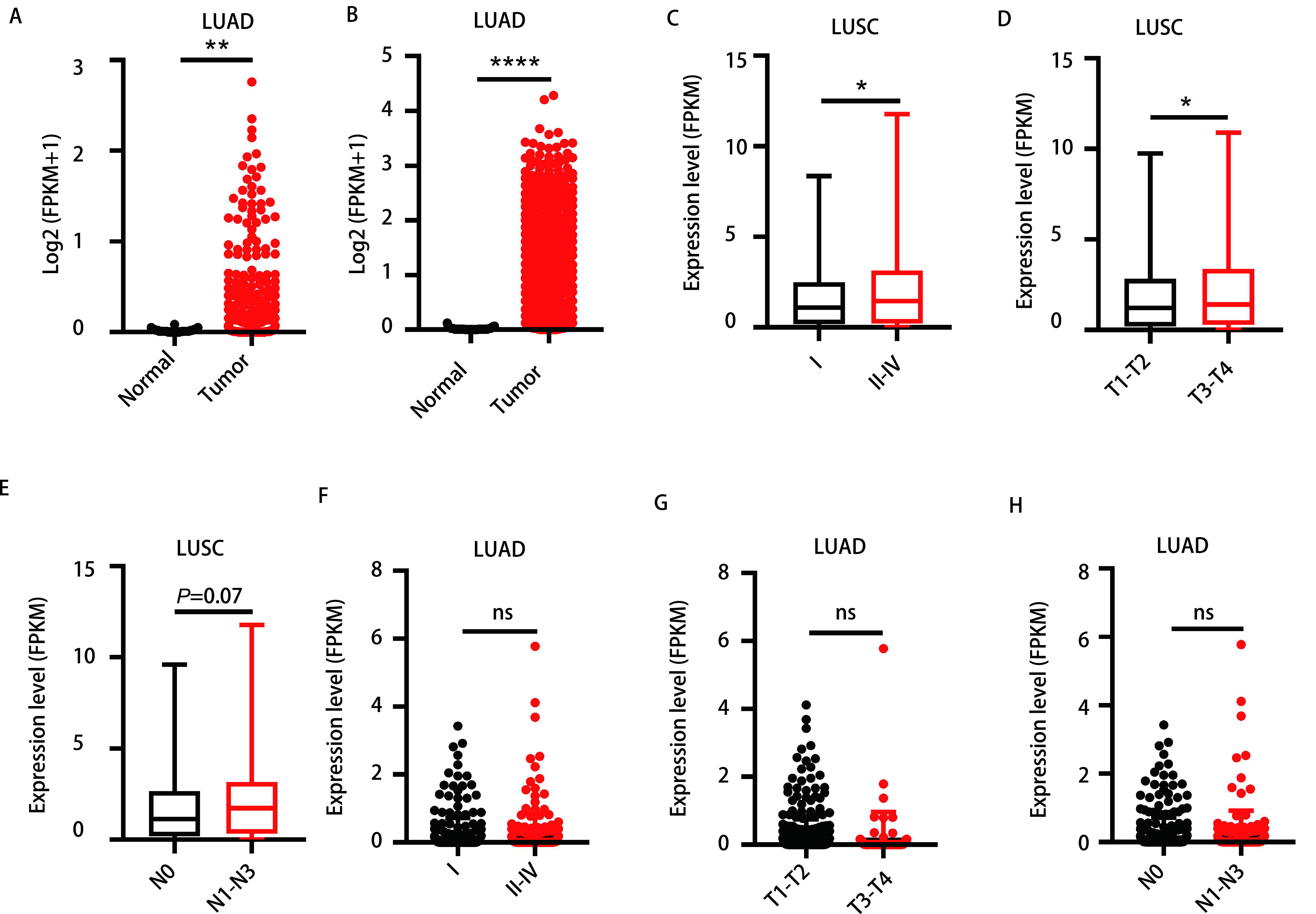
LINC00668在LUSC中表达量升高且与患者TNM分期显著相关。A：TCGA数据库中LINC00668在LUAD组织和正常组织中的表达情况; B：TCGA数据库中LINC00668在LUSC组织和正常组织中的表达情况; C：TCGA数据库中LINC00668在不同肿瘤分期LUSC患者组织中的表达情况; D：TCGA数据库中LINC00668在不同T分期LUSC患者组织中的表达情况; E：TCGA数据库中LINC00668在不同N分期LUSC患者组织中的表达情况; F：TCGA数据库中LINC00668在不同肿瘤分期LUAD患者组织中的表达情况; G：TCGA数据库中LINC00668在不同T分期LUAD患者组织中的表达情况; H：TCGA数据库中LINC00668在不同N分期LUAD患者组织中的表达情况。^*^P < 0.05，^**^P < 0.01，^****^P < 0.000, 1，ns：无统计学意义。 LINC00668 expression was up-regulated in LUSC patients and was correlated to advanced TNM stage. A: The expression level of LINC00668 in LUAD and normal tissues based on TCGA database; B: The expression level of LINC00668 in LUSC and normal tissues based on TCGA database; C: The expression level of LINC00668 in LUSC samples with different tumor stages based on TCGA database; D: The expression level of LINC00668 in LUSC samples with different T stages based on TCGA database; E: The expression level of LINC00668 in LUSC samples with different N stages based on TCGA database; F: The expression level of LINC00668 in LUAD samples with different tumor stages based on TCGA database; G: The expression level of LINC00668 in LUAD samples with different T stages based on TCGA database; H: The expression level of LINC00668 in LUAD samples with different N stages based on TCGA database. ^*^P < 0.05, ^**^P < 0.01, ^****^P < 0.000, 1, ns: not significant. TNM: tumor-node-metastasis.

随后我们分别在LUSC及LUAD中进一步分析了LINC00668的表达量与TNM分期的关系。结果显示，在LUSC中，LINC00668的表达量和肿瘤分期密切相关，相较于早期(Ⅰ期)，其表达量在中晚期(Ⅱ期-Ⅳ期)LUSC中明显升高(1.77±0.13 *vs* 2.16±0.15, *P* < 0.05)(图 1C); 同时，相较于T1-T2，LINC00668在T分期晚期(T3-T4)的LUSC中表达量也显著升高(1.88±0.10 *vs* 2.38±0.28, *P* < 0.05)(图 1D)。虽然其表达量在N分期的早期及中晚期的LUSC中无明显差异，但是有明显趋势显示其在N分期中晚期(N1-N3)时表达量增高(1.85±0.12 *vs* 2.23±0.18, *P*=0.07)(图 1E)。在LUAD中，LINC00668的表达量与肿瘤的TNM分期无明显关系(图 1F-图 1H)。

接下来，我们又以中位表达量为界将LUSC患者分为LINC00668高表达或低表达组，分别探索其与患者年龄、性别、TNM分期及吸烟情况的关系，结果显示，LINC00668高表达倾向于男性、晚期及吸烟患者(表 4)。

**表 4:** TCGA数据库中LINC00668表达量高低两组的LUSC患者临床特征[*n*(%)] Clinical characteristics of LUSC patients with high or low LINC00668 expression level in TCGA database [*n*(%)]

Clinical factors	TCGA dataset - LUSC		*P*
	Low group	High group	
Age (yr)			0.106
≤65	86 (35.1)	103 (42.2)	
> 65	159 (64.9)	141 (57.8)	
Gender			0.024
Female	75 (30.4)	53 (21.5)	
Male	172 (69.6)	194 (78.5)	
Stage			0.039
Ⅰ	127 (52.0)	115 (46.7)	
Ⅱ	66 (27.0)	92 (37.4)	
Ⅲ/Ⅳ	51 (21.0)	39 (15.9)	
T stage			0.813
T1	54 (21.9)	60 (24.3)	
T2	146 (59.1)	141 (57.1)	
T3-T4	47 (19.0)	46 (18.6)	
N stage			0.583
0	163 (66.8)	153 (62.7)	
1	57 (23.4)	70 (28.7)	
2	21 (8.6)	19 (7.8)	
3	3 (1.2)	2 (0.8)	
Smoking status			0.003
Non or ever	187 (78.2)	157 (66.0)	
Current	52 (21.8)	81 (34.0)	

### 2.2 LINC00668与吸烟密切相关且影响LUSC吸烟患者的预后

我们利用TCGA数据库中OS数据进一步探索了LINC00668的表达和生存的关系，结果显示，无论在LUSC或LUAD中，LINC00668的表达量与患者的OS无明显相关(*P* > 0.05)(图 2A，图 2B)。

**图 2:**
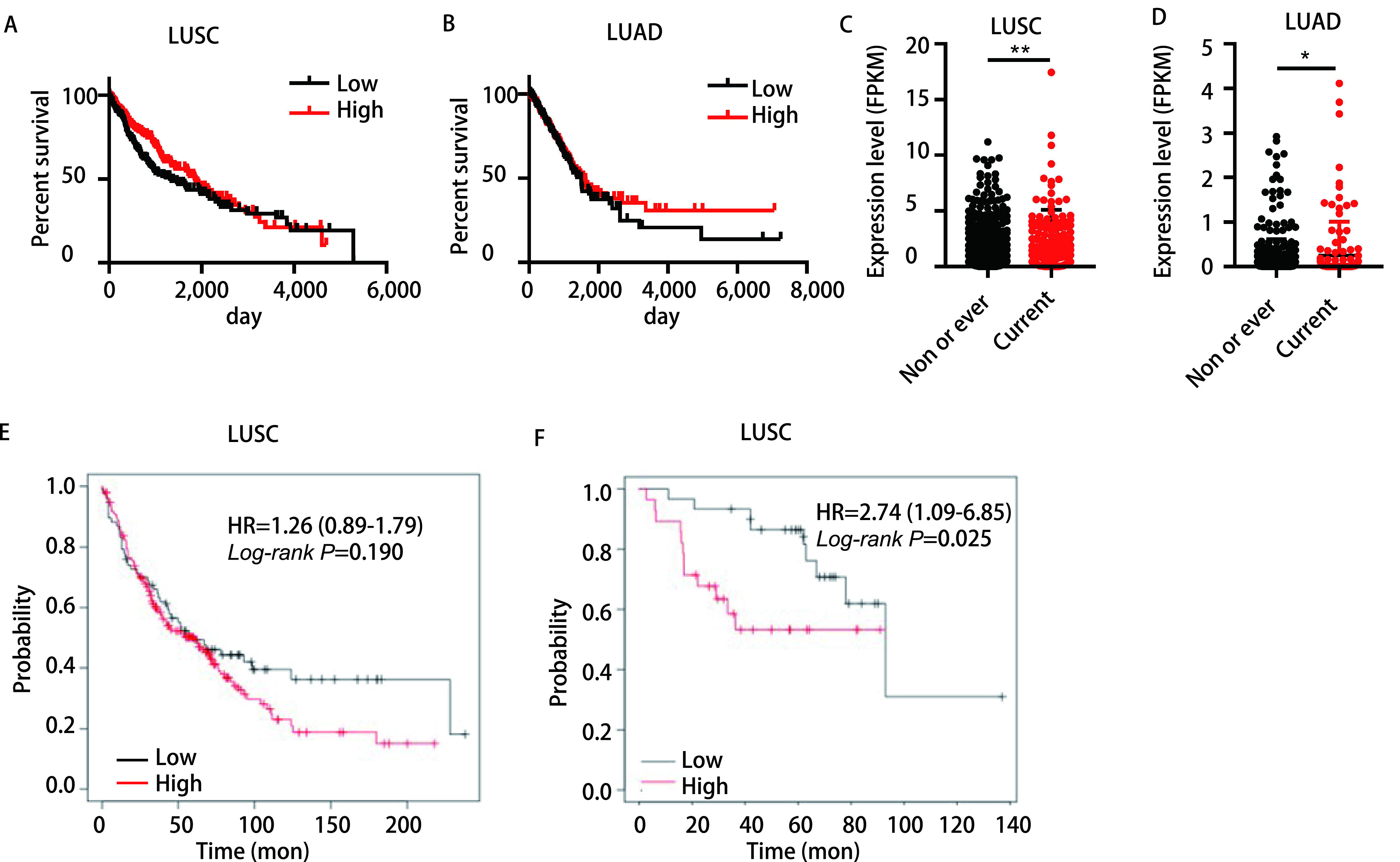
LINC00668与吸烟密切相关且影响LUSC吸烟患者的预后。A：*Kaplan-Meier*生存曲线显示TCGA数据库中LUSC患者LINC00668表达量高低两组(以LINC00668中位表达量为界)的总生存期(overall survival, OS)的情况; B：*Kaplan-Meier*生存曲线显示TCGA数据库中LUAD患者LINC00668表达量高低两组(以LINC00668中位表达量为界)的OS的情况; C：TCGA数据库中LINC00668在LUSC吸烟和未吸烟患者组织中的表达情况; D：TCGA数据库中LINC00668在LUAD吸烟和未吸烟患者组织中的表达情况; E：Kmplot中LUSC患者LINC00668表达量高低两组OS的情况; F：Kmplot中LINC00668在吸烟LUSC患者中表达量高低两组OS的情况。^*^P < 0.05, ^**^P < 0.01。 LINC00668 expression is strongly associated with smoking and has impact on the prognosis of patients with smoking history. A: *Kaplan-Meier* survival curve of overall survival (OS) among LUSC patients with low or high expression of LINC00668 (median expression of LINC00668 as cut-off) based on TCGA database; B: *Kaplan-Meier* survival curve of OS among LUAD patients with low or high expression of LINC00668 (median expression of LINC00668 as cut-off) based on TCGA database; C: The expression level of LINC00668 in LUSC patients according to their different smoking status based on TCGA database; D: The expression level of LINC00668 in LUAD patients according to their different smoking status based on TCGA database; E: *Kaplan-Meier* survival curve of OS among LUSC patients with low or high expression of LINC00668 in Kmplot; F: *Kaplan-Meier* survival curve of OS among LUSC smokers with low or high expression of LINC00668 in Kmplot. ^*^P < 0.05, ^**^P < 0.01.

吸烟是肺癌的独立危险因素，且参与并促进肺癌的发生发展。接下来，我们进一步探索了LINC00668的表达量和吸烟情况的关系。结果显示，无论在LUSC或LUAD中，LINC00668在吸烟患者中的表达量均明显升高(LUSC: 1.84±0.12 *vs* 2.46±0.23; LUAD: 0.16±0.02 *vs* 0.29±0.07)，差异具有统计学意义(*P* < 0.05)(图 2C，图 2D)。

*Kaplan-Meier* Plotter(Kmplot)网站囊括了CaBIG(http://cabig.cancer.gov/)数据库、TCGA数据库及GEO数据库中肺癌的数据，可以扩大样本量更好地分析基因与生存的关系。有趣的是，在LUSC中，LINC00668的表达量仍然与患者的OS无明显相关(*P* > 0.05)(图 2E); 然而，LINC00668的表达量却与吸烟LUSC患者的OS显著相关，LINC00668高表达的吸烟肺鳞癌患者的生存期明显低于低表达的吸烟患者[HR: 2.74 (1.09-6.85), *P* < 0.05](图 2F)。

LINC00668的表达量与LUSC患者的临床分期相关; 吸烟是LUSC的高危因素之一，LINC00668表达量在吸烟患者中显著升高，且与吸烟LUSC患者的OS显著相关。以上结果提示，与LUAD相比，LINC00668与LUSC关系更加密切。

### 2.3 LINC00668在LUSC细胞系及组织中表达量升高

为了验证我们的猜想，我们检测了LINC00668在正常支气管上皮及多种LUAD或LUSC细胞系中的表达量。相较于正常支气管上皮细胞HBE，LINC00668在三株LUSC细胞系H2170、H226、H520中表达量明显升高，差异具有统计学意义(*P* < 0.05); 但是，其在LUAD细胞系A549和H1299中的表达量无显著升高(*P* > 0.05)(图 3A)。

**图 3:**
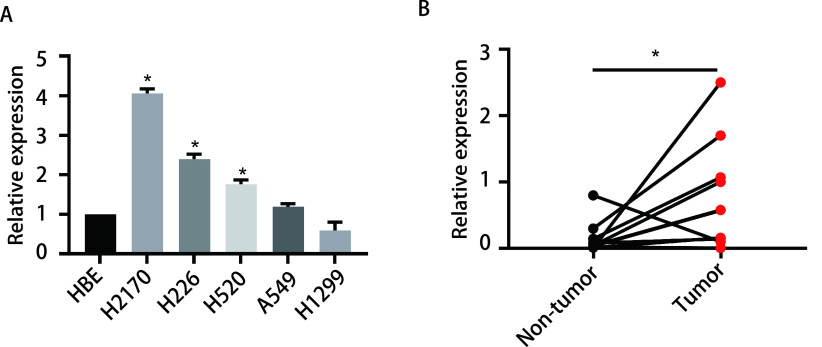
LINC00668在肺鳞癌细胞系和组织中表达量升高。A：qRT-PCR检测LINC00668在不同肺腺癌和肺鳞癌细胞系中的表达情况，每种细胞系设置3个重复孔; B：qRT-PCR检测LINC00668在10对肺鳞癌组织及其癌旁组织中的表达情况。^*^P < 0.05，每组实验重复3次。 LINC00668 expression was increased in LUSC cell lines and tissues. A: The expression level of LINC00668 in different LUAD and LUSC cell lines according to qRT-PCR assay, each cell line has three duplicates; B: The expression level of LINC00668 in LUSC tissues and corresponding adjacent non-tumor tissues according to qRT-PCR assay. ^*^P < 0.05, each experiment repeated three times.

qRT-PCR结果显示LINC00668在肿瘤组织中的表达量明显高于其相应的癌旁组织，差异具有统计学意义(*P* < 0.05)(图 3B)。

### 2.4 敲低LINC00668不影响肺鳞癌细胞的增殖，但抑制其转移侵袭

我们选择LINC00668表达量最高的LUSC细胞系H2170进行下一步功能实验的探索。转染后荧光镜下图片显示转染效率 > 70%(图 4A)。qRT-PCR结果显示转染质粒shRNA1和shRNA2的H2170细胞表达LINC00668的量明显降低，差异具有统计学意义(*P* < 0.05)(图 4B)。因此，在H2170细胞系中成功构建了有效的LINC00668敲低模型。

**图 4:**
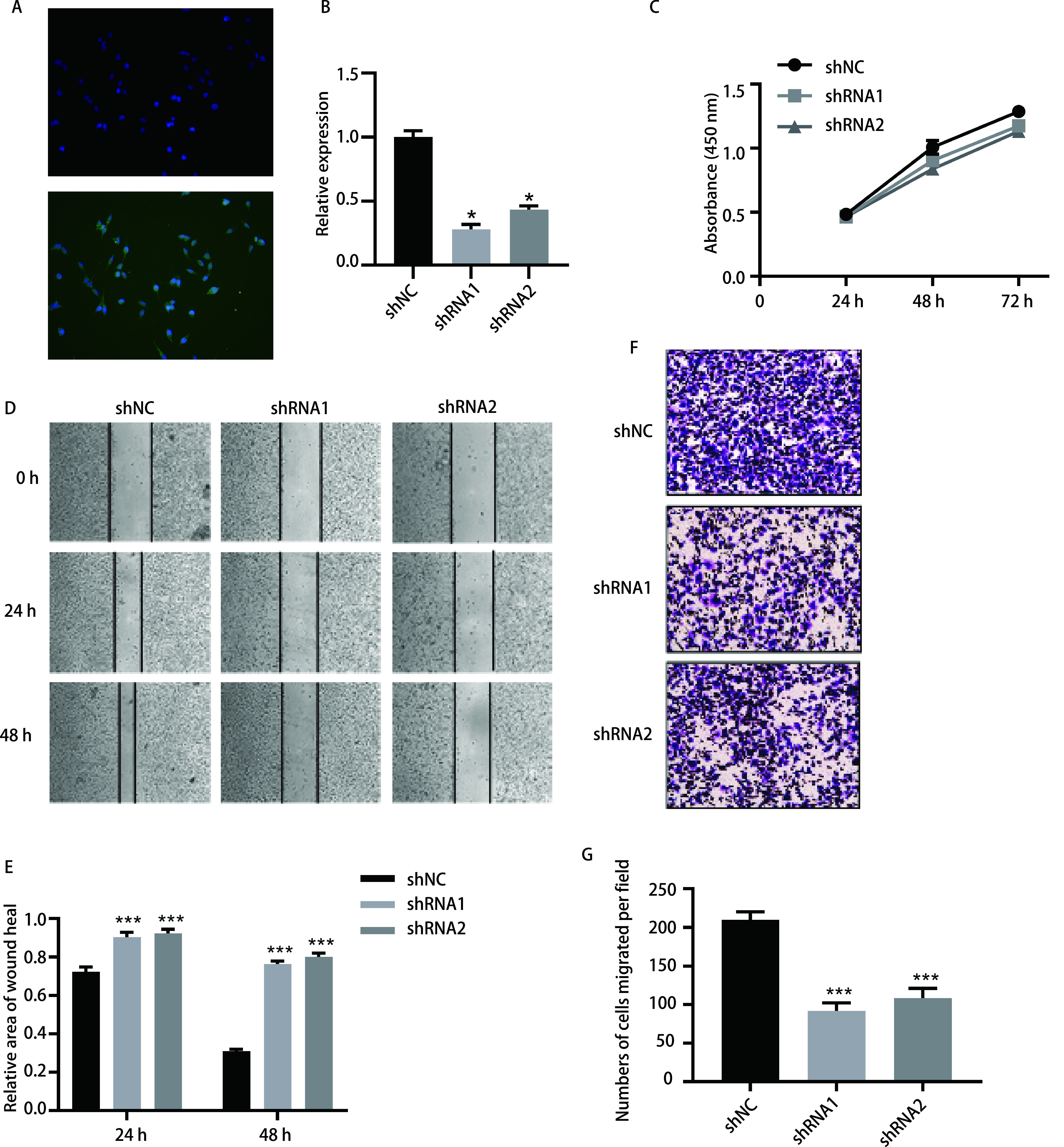
在肺鳞癌细胞系H2170中下调LINC00668可抑制细胞的转移和侵袭。A：荧光显微镜下显示H2170细胞转染后的荧光携带情况; B：qRT-PCR检测显示LINC00668在转染质粒shRNA的H2170细胞系中表达量降低; C：CCK-8试验显示在H2170细胞中下调LINC00668不影响细胞的增殖; D：划痕试验显示在H2170细胞中下调LINC00668抑制细胞迁移; E：划痕试验划痕面积的量化(面积较0 h的相对大小); F：Transwell试验显示在H2170细胞中下调LINC00668抑制细胞侵袭; G：Transwell试验侵袭到小室下层膜细胞数的量化。^*^P < 0.05，^***^P < 0.001，每组实验重复3次。 Downregulation of LINC00668 in LUSC cell line H2170 suppressed cell migration and invasion. A: Fluorescence microscopy showed the transfection efficiency of H2170 cells; B: qRT-PCR showed that the expression of LINC00668 in H2170 cells was significantly reduced after transfection with shRNAs; C: CCK-8 assay showed knockdown of LINC00668 in H2170 cells does not affect cell proliferation; D: Wound-healing assay showed knockdown of LINC00668 in H2170 cells inhibited cell migration; E: Quantification of wound area in wound-healing assay (normalized by the area of 0 h); F: Transwell assay showed knockdown of LINC00668 in H2170 cells suppressed cell invasion; G: Quantification of the number of cells invaded into down chambers. ^*^P < 0.05, ^***^P < 0.001, each experiment repeated three times.

为了探索LINC00668在H2170细胞增殖中的作用，我们进行了CCK-8实验，分别在铺板后24 h、48 h及72 h检测了三组细胞的吸光度值，结果显示，三组并无明显差异(图 4C)。以上结果提示，在H2170细胞中敲低LINC00668并不影响细胞的增殖。

为了探索LINC00668对H2170细胞迁移侵袭的影响，我们首先进行了划痕实验，结果显示，划痕后24 h和48 h，LINC00668敲低组shRNA1和shRNA2的划痕区域面积明显大于对照组(*P* < 0.05)(图 4D，图 4E)。Transwell小室侵袭实验显示，LINC00668敲低组shRNA1和shRNA2穿到小室下面的细胞数明显少于对照组(210±6 *vs* 92±6 *vs* 108±7, *P* < 0.05)(图 4F，图 4G)。以上结果提示，敲低LINC00668显著抑制了H2170肺鳞癌细胞的迁移和侵袭能力。

## 3 讨论

肺癌仍是人类最常见的恶性肿瘤之一和主要的公共卫生问题。因此，发现新的分子诊断指标和特异性的治疗靶点十分关键。近年来，随着对LncRNAs的生物学功能及作用机制的深入研究，越来越多的证据指出其在肿瘤的发生发展过程中扮演着重要角色。现已发现多种LncRNAs参与NSCLC的发生、发展及耐药等复杂的生物学过程^[20]^。在本研究中，通过对公共数据库TCGA的分析，结合体外的生物学功能试验，我们发现LINC00668是一个与吸烟相关且影响LUSC细胞转移侵袭的特异性LncRNA，可以通过抑制LINC00668减少LUSC的转移。

NSCLC是一种异质性疾病，根据标准病理学分类，可分为LUAD和LUSC两大亚类。随着二代测序(second-generation sequencing, NGS)技术的进步和肺癌精准治疗的发展，LUAD和LUSC在基因组层面的差异逐渐明了，两者有着明显不同的突变模式^[21-23]^。例如，已成功研发出有效的靶向药物的表皮生长因子受体(epidermal growth factor receptor, *EGFR*)、间变性淋巴瘤激酶(anaplastic lymphoma kinase, *ALK*)和c-ros肉瘤致癌因子受体酪氨酸激酶(ROS proto-oncogene 1, receptor tyrosine kinase, *ROS1*)等驱动突变常发生在LUAD中，而这些驱动突变在LUSC中的发生率则较低^[24, 25]^，提示相同的分子在LUAD或LUSC中可能扮演着不同的角色。以往的研究^[17]^显示，LINC00668在NSCLC中表达量增高，在TNM分期晚、伴有淋巴结转移的患者中表达量较高，同时其表达量增高与患者的不良预后密切相关。但是该研究并没有分别探索LINC00668的表达量与LUAD或LUSC两种肺癌亚型患者的临床特征的关系。有趣的是，我们的研究发现，LINC00668虽然在LUAD和LUSC中的表达量均明显升高，但与LUAD不同的是，在LUSC中，其表达量和患者的TNM分期密切相关。更值得一提的是，该LncRNA与吸烟关系密切，吸烟又是肺鳞癌的重要特征之一^[26]^，同时它还是吸烟LUSC患者的预后指标，这些特点都指出LINC00668可能是参与LUSC发生发展的特异性LncRNA。以前也有报道指出LINC00668参与鳞癌细胞的增殖、侵袭或转移。Zhang等^[27]^报道LINC00668在口腔鳞状细胞癌中表达量显著升高; 同时，其通过和miR-297相互作用促进口腔鳞状细胞的增殖。Zhao等^[28]^通过对GEO数据库中喉鳞状细胞癌的芯片分析，发现LINC00668在喉鳞状细胞癌中表达量显著升高，且与患者的临床分期、病理分化程度及淋巴结转移情况相关。同时，体外实验敲低LINC00668可有效抑制喉鳞状细胞癌细胞的增殖、侵袭和转移。

肺癌在最初诊断时常伴随侵袭和转移，是造成肺癌死亡的主要原因^[29]^。在本研究中，我们发现在肺鳞癌细胞系H2170中敲低LINC00668并不影响细胞增殖，但是明显抑制了细胞的侵袭和转移。在另一项关于NSCLC的研究^[17]^中，研究者发现敲低LINC00668可以促进LUAD细胞系A549和H1299的凋亡从而抑制细胞增殖。LINC00668在不同肿瘤细胞中的生物学功能有所不同。在乳腺癌中，敲低LINC00668可有效促进凋亡并抑制细胞周期，从而抑制肿瘤细胞增殖^[13]^; 同时，另有一篇文献^[14]^报道其通过调节SND1影响乳腺癌细胞的干细胞性和侵袭转移。在胃癌和肝癌中，LINC00668通过促进上皮-间质转化(epithelial-mesenchymal transition, EMT)影响肿瘤细胞的侵袭和转移^[15, 30]^。在结肠癌中，LINC00668通过和miR-188-5p相互作用调节USP47促进肿瘤的发生和进展。

当然，本研究也有很多不足之处。第一，我们仅在10对LUSC组织及其配对的癌旁组织中检测了LINC00668的表达量，后续可以增加样本量进行验证并探讨LINC00668表达量和患者临床特征的关系; 第二，本研究的体外实验只运用了一种细胞系，可增加细胞系种类进一步验证LINC0068在LUSC中的生物学功能; 第三，我们并没有深入探究LINC00668影响LUSC细胞侵袭转移的具体机制，这也是接下来我们的重点工作内容。

综上所述，我们的研究初步证明了LINC00668在LUSC中高表达，与LUSC患者的肿瘤TNM分期密切相关，同时它还是吸烟LUSC患者的预后指标。体外细胞中敲低LINC00668可有效降低LUSC细胞的侵袭转移能力。以上结果提示LINC00668可能与LUSC发生发展密切相关，通过抑制LINC00668可以在一定程度上减少LUSC的转移。
